# Prevalence of *aac(6')-Ib-cr *plasmid-mediated and chromosome-encoded fluoroquinolone resistance in *Enterobacteriaceae *in Italy

**DOI:** 10.1186/1757-4749-3-12

**Published:** 2011-08-09

**Authors:** Ilaria Frasson, Antonietta Cavallaro, Cristina Bergo, Sara N Richter, Giorgio Palù

**Affiliations:** 1 Department of Histology, Microbiology and Medical Biotechnologies, University of Padua, via Gabelli 63, 35121 Padua, Italy; 2 Azienda Ospedaliera of Padua, Microbiology and Virology Unit, via Giustiniani 2, 35121 Padua, Italy

**Keywords:** *aac(6')-Ib-cr*, fluoroquinolones, plasmid-mediated resistance, Gram-negative

## Abstract

The spread of *aac(6')-Ib-cr *plasmid-mediated quinolone resistance determinants was evaluated in 197 enterobacterial isolates recovered in an Italian teaching hospital. The *aac(6')-Ib-cr *gene was found exclusively in *Escherichia coli *strains. The gene was located on a plasmid which presented additional ESBL genes. Most of the clinical strains were clonally related and displayed three point mutations at the topoisomerase level which conferred high resistance to fluoroquinolones.

## Findings

The aminoglycoside acetyltransferase *Aac(6')-Ib-cr *variant, an enzyme usually encoded by a plasmid-borne gene, extends its drug targets to include fluoroquinolones (FQs) in addition to aminoglycosides. It is characterized by amino acid changes at codon 102 (Trp→Arg) and codon 179 (Asp→Tyr). The *Aac(6')-Ib-cr *protein is able to specifically acetylate hydrophilic FQs presenting a free piperazinyl amine (i.e. ciprofloxacin and norfloxacin) [1].

The *aac(6')-Ib-cr *gene has spread rapidly among *Enterobacteriaceae*, and although only conferring a low-level resistance, it may create an environment facilitating the selection of more highly resistant determinants, especially those harbouring topoisomerase mutations. This fact is particularly worrisome at the nosocomial level, where *aac(6')-Ib-cr *containing strains should be promptly detected and treated with non-hydrophilic FQs, such as levofloxacin or ofloxacin, or other classes of antibiotics to prevent high-level resistance onset and spread.

In this work we determined the prevalence of the *aac(6')-Ib-cr *gene variant among clinical isolates of *Enterobacteriacea *collected at the teaching Hospital of Padua, Italy. In the time period of March-May 2008, 197 non-duplicate clinical isolates were collected. These displayed the whole range of MIC of ciprofloxacin; in particular, 104 samples were susceptible (MIC ≤ 1), 35 intermediate (1 < MIC < 4), and 58 resistant (MIC ≥ 4) to ciprofloxacin. Exact MIC values were measured by means of E-test strips (AB Biodisk, Solna, Sweden). Bacterial isolates were: 145 *E. coli*, 38 *K. pneumoniae*, 5 *Proteus mirabilis*, 5 *Enterobacter aerogenes*, 2 *Enterobacter cloacae *and 2 *Citrobacter freundii*. Sample identity and results are reported in Table 1.

**Table 1:** Properties of *aac(6')-Ib*-positive clinical isolates and transformant and transconjugant strains

Isolate ID	Bacterial species	*qnr gene*	*aac(6') -Ib gene*	MIC (mg/L)					GyrA	ParC	ESBL^i ^phen	ESBL genotypic	ERIC
									
				NA^d^	CPF^e ^	OFX^f^	LVF^g^	MXF^h^					
6	*Escherichia coli*	*cr*	-	≥ 4	≥ 4	≥ 16	≥ 16	≥ 16	S83L, D87N	E84V	-	-	1

6**T**^a^	*E. coli TOP10*	*cr*	-	0.008	0.008	0.016	0.004	0.006	-	-	nd	-	

13	*E. coli*	*cr*	-	≥ 4	≥ 4	≥ 16	≥ 16	≥ 16	S83L, D87N	E84V	-	CTX-M-1	1

13**C**^b^	*J53AzKanNaR*	*cr*	-	0.064	0.064	0.047	0.047	0.023	-	-	nd	CTX-M-1	

13**T**	*E. coli TOP10*	*cr*	-	0.008	0.008	0.016	0.004	0.006	-	-	nd	CTX-M-1	

19	*E. coli*	*non-cr*	-						nd	nd	nd	nd	

23	*E. coli*	*cr*	-	≥ 4	≥ 4	≥ 16	≥ 16	≥ 16	S83L, D87N	E84V	X	CTX-M-1+TEM-1	2

23**C**	*J53AzKanNaR*	*cr*	-	0.047	0.047	0.047	0.047	0.032	-	-	nd	CTX-M-1+TEM-1	

23**T**	*E. coli TOP10*	*cr*	-	0.004	0.004	0.016	0.004	0.006	-	-	nd	CTX-M-1+TEM-1	

37	*E. coli*	*cr*	-	≥ 4	≥ 4	≥ 16	≥ 16	≥ 16	S83L, D87N	E84V	X	CTX-M-1+TEM-1	1

37**T**	*E. coli TOP10*	*cr*	-	0.006	0.006	0.016	0.004	0.006	-	-	nd	CTX-M-1+TEM-1	

39	*E. coli*	*cr*	-	≥ 4	≥ 4	≥ 16	≥ 16	≥ 16	S83L, D87N	E84V	X	CTX-M-1	3

39**T**	*E. coli TOP10*	*cr*	-	0.006	0.006	0.016	0.004	0.006	-	-	nd	CTX-M-1	

40	*E. coli*	*cr*	-	≥ 4	≥ 4	≥ 16	≥ 16	≥ 16	S83L, D87N	E84V	X	CTX-M-1+TEM-1	1

40**T**	*E. coli TOP10*	*cr*	-	0.006	0.006	0.016	0.004	0.006	-	-	nd	CTX-M-1+TEM-1	

44	*E. coli*	*cr*	-	0.047	0.047	0.032	0.047	0.023	-	-	X	CTX-M-1	3

44**C**	*J53AzKanNaR*	*cr*	*-*	0.047	0.047	0.047	0.047	0.023	-	-	nd	CTX-M-1	

44**T**	*E. coli TOP10*	*cr*	-	0.006	0.006	0.016	0.004	0.006	-	-	nd	CTX-M-1	

51	*E. coli*	*cr*	-	≥ 4	≥ 4	≥ 16	≥ 16	≥ 16	S83L, D87N	E84V	X	CTX-M-1	1

51**T**	*E. coli TOP10*	*cr*	-	0.006	0.006	0.016	0.004	0.006	-	-	nd	CTX-M-1	

52	*E. coli*	*cr*	-	≥ 4	≥ 4	≥ 16	≥ 16	≥ 16	S83L, D87N	E84V	X	CTX-M-1	1

52**T**	*E. coli TOP10*	*cr*	-	0.004	0.004	0.016	0.004	0.006	-	-	nd	CTX-M-1	

53	*E. coli*	*cr*	-	≥ 4	≥ 4	≥ 16	≥ 16	≥ 16	S83L, D87N	E84V	X	CTX-M-1	1

53**T**	*E. coli TOP10*	*cr*	-	0.004	0.004	0.016	0.004	0.006	-	-	nd	CTX-M-1	

111	*Klebsiella pneumoniae*	*non-cr*	*qnrB19*	1.5	1.5	4	2	nd	nd	nd	X	TEM-1+SHV-12	

128	*K. pneumoniae*	*non-cr*	-	1.5	1.5	3	2	2	nd	nd	nd	nd	

137	*K. pneumoniae*	*non-cr*	*qnrB19*	1	1	4	1.5	1.5	nd	nd	X	TEM-1	

143	*K. pneumoniae*	*non-cr*	*qnrB19*	1.5	1.5	4	1.5	1.5	nd	nd	-	TEM-150+SHV-12	

144	*K. pneumoniae*	*non-cr*	*qnrB19*	1.5	1.5	6	1.5	1.5	nd	nd	X	TEM-150	

164	*E. coli*	*cr*	-	≥ 4	≥ 4	≥ 16	≥ 16	≥ 16	S83L, D87N	E84V	X	CTX-M-1+TEM-1	1

164**T**	*E. coli TOP10*	*cr*	-	0.004	0.004	0.016	0.004	0.006	-	-	nd	CTX-M-1	

175	*E. coli*	*cr*	-	≥ 4	≥ 4	nd	≥ 8	≥ 8	S83L, D87N	E84V	X	CTX-M-1	1

175**T**	*E. coli TOP10*	*cr*	-	0.006	0.006	0.016	0.004	0.006	-	-	nd	CTX-M-1	

176	*K. pneumoniae*	*non-cr*	*qnrB19*	1	1	2	1.5	≥ 8	nd	nd	-	TEM-150	

178	*E. coli*	*cr*	-	≥ 4	≥ 4	nd	≥ 8	≥ 8	S83L, D87N	E84V	X	CTX-M-1+TEM-1	1

178**T**	*E. coli TOP10*	*cr*	-	0.006	0.006	0.016	0.004	0.006	-	-	nd	CTX-M-1+TEM-1	

180	*K. pneumoniae*	*non-cr*	*-*	0.25	0.25	0.75	0.25	0.38	nd	nd	X	nd	

182	*E. coli*	*cr*	-	≥ 4	≥ 4	≥ 8	≥ 8	≥ 8	S83L, D87N	E84V	X	CTX-M-1	1

182**T**	*E. coli TOP10*	*cr*	-	0.006	0.006	0.016	0.004	0.006	-	-	nd	CTX-M-1	

183	*Enterobacter aerogenes*	*non-cr*	-	0.75	0.75	2	1	0.75	nd	nd	nd	nd	

184	*E. coli*	*cr*	-	≥ 4	≥ 4	nd	≥ 8	≥ 8	S83L, D87N	E84V	X	CTX-M-1+TEM-1	1

184**T**	*E. coli TOP10*	*cr*	-	0.006	0.006	0.016	0.004	0.006			nd	CTX-M-1+TEM-1	

185	*E. coli*	*cr*	-	≥ 4	≥ 4	nd	≥ 8	≥ 8	S83L, D87N	E84V	X	CTX-M-1+TEM-1	1

185**T**	*E. coli TOP10*	*cr*	-	0.006	0.006	0.016	0.004	0.006	-	-	nd	CTX-M-1+TEM-1	

	*E. coli TOP10*	*-*	-	0.016	0.016	0.047	0.047	0.023	-	-	nd	-	

	*J53AzKanR*	*-*	-	0.002	0.002	0.016	0.004	0.006	-	-	nd	-	

^a^**T **= tranformant strain; ^b^**C**: transconjugant strain. ^**d**^NA = nalidixic acid, ^**e**^CPF = ciprofloxacin, ^**f**^OFX = ofloxacin, ^**g**^LVF = levofloxacin, ^**h**^MXF = moxifloxacin. ^**i**^ESBL phen = ESBL phenotypic determination.

nd = not determined

- = negative

Resistance ranges: nalidixic acid S ≤ 16 mg/L, R ≥ 32 mg/L; ciprofloxacin S ≤ 1 mg/L, I = 2 mg/L, R ≥ 4 mg/L; ofloxacin, levofloxacin, moxifloxacin S ≤ 2 mg/L, I = 4 mg/L, R ≥ 8 mg/L.

The presence of the *aac(6')-Ib *or *aac(6')-Ib-cr *genes was assessed by PCR amplification and subsequent sequencing with primers: aacF 5'-ATGACTGAGCATGACCTTG-3'; aacR 5'-AACCATGTACACGGCTGG-3'; aacSEQ 5'-CGTCACTCCATACATTGCAA-3' [2].

Twenty-five samples out of 197 (13%) were positive for the *aac(6')-Ib *gene and of these 16 (8%) displayed the *aac(6')-Ib-cr *variant. In particular, *aac(6')-Ib-cr *was found exclusively in *E. coli*, while *aac(6')-Ib *was present mostly in *K. pneumoniae *(8 *K. pneumoniae*, 1 *E. coli*). Out of the 16 *aac(6')-Ib-cr*-positive samples, 15 were collected from urine and 1 from skin; 13 were from inpatients (81%) and 3 from outpatients.

All *aac(6')-Ib *were tested for other plasmid-mediated quinolone-resistence genes, i.e. *qnr *and *qepA*, by PCR amplification and sequencing using published procedures [3]. None of these was found in the *aac(6')-Ib-cr*-positive samples, while 5 out of 9 *aac(6')-Ib*-positive strains presented the *qnrB19 *gene, indicating that just one of the reported plasmid-encoded mechanisms of FQ resistance was acquired/maintained in the clinical isolates.

The presence of mechanisms of chromosomial resistance to FQ was assessed on *aac(6')-Ib-cr*-positive strains. The genotypic analysis, performed with universal primers [4], revealed that all samples, but #44, coded for two mutations in GyrA (S83L, D87N) and one mutation in ParC (E84V). Phenotypic analysis of resistance to nalidixic acid and FQs confirmed the above results: 15 samples were resistant to all tested drugs, while one (#44) was fully susceptible.

The presence of resistance to other classes of antibiotic was next assessed. Genotypic analysis of ESBL and AmpC genes was performed by PCR amplification using specific primers for the detection of ESBL, i.e. bla_SHV_, bla_TEM_, bla_CTX-M-1, 2, 8, 9_, and AmpC genes, i.e. *bla*_MOX-1, 2_, *bla*_CMY-1-11_, *bla*_LAT-1-4_, *bla*_BIL-1_, *bla*_DHA-1, 2_, *bla*_ACC_, *bla*_MIR-1T_, *bla*_ACT-1_, *bla*_FOX-1, 5b _[5-8]. Fifteen out of 16 samples presented the CTX-M-1 gene, which was coupled with TEM-1 in 7 strains. No other *bla *genes, or AmpC were detected. ESBL production, phenotypically measured according to the CLSI M100-S18 and M2-A9 documents [9], was detected in 14 out of 16 samples. Accordingly, most of the samples were resistant to β-lactams, while they retained susceptibility to amikacin, imipenem, piperacillin/tazobactam; an equal number of samples resistant and susceptible to gentamicin, trimethoprim/sulfamethoxazole, ceftazidime, cefepime, aztreonam and tetracycline was detected (data not shown).

**Figure 1:**
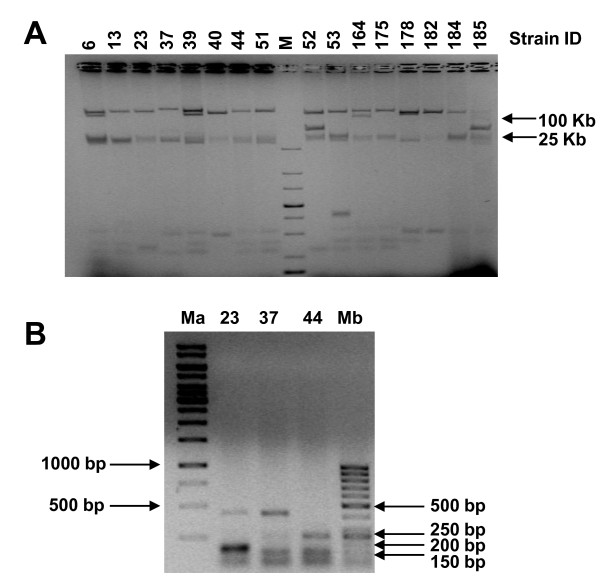
***aac(6')-Ib-cr*-positive strain analysis**. A) Analysis of plasmids extracted from *aac(6')-Ib-cr*-positive strains by the Kieser method. Plasmids were run on 0.7% TAE agarose gel at 50 V for 5 h. The numbers above each lane indicate the clinical strains ID. M stands for marker. B) Clonal relationship based on the repetitive element PCR fingerprinting method, using "enterobacterial repetitive intergenic consensus" (ERIC) primers. Amplified PCR products representative of the three identified subgroups were loaded on 1% TAE agarose gels and run at 100 V for 2 h. Both gels were stained with ethidium bromide.

Plasmid DNA was extracted from *aac(6')-Ib-cr*-positive strains and run on agarose gel, to confirm the presence of the *aac(6')-Ib-cr *gene on a mobile element: all samples presented two main electrophoretic band corresponding to > 100 Kbp and 25 Kbp (Figure 1A). In each case the *aac(6')-Ib-cr *gene was located in the > 100 Kbp band, as demonstrated by PCR gene amplification, using DNA extracted from each band as template. Plasmid localization of the *aac(6')-Ib-cr *gene was further confirmed by successful transformation into *E. coli *Top10 strain of all 16 sample-plasmid DNA, extracted according the Kieser protocol [10]. Transferability of the resistance gene of three clinical isolates was tested by conjugation in a kanamycin/sodium azide resistant *E. coli *J53 strain [3]. All three samples were successfully conjugated. The presence of the *aac(6')-Ib-cr *was confirmed by PCR in both transformants and transconjugants. In addition, the ESBL genes previously detected in the clinical isolate were always found on the same plasmid as the *aac(6')-Ib-cr *gene: just in one case (sample #164) TEM-1 was not co-transformed along with *aac(6')-Ib-cr *and CTX-M-1.

MIC analysis of FQs in transformants and tranconjugants compared to the wild type isolates showed a drastic decrease in the MIC values of all tested antibiotics. However, MIC of ciprofloxacin increased of 2-4 times in transformants and transconjugats, compared to the wild type recipient strains, *E. coli *Top10 and J53. These results are in line with the notion that the *aac(6')-Ib-cr *alone does not confer high level resistance to the drugs, but it stimulates chromosomal mutations on the FQ targets, i.e. gyrase and topoisomerase IV, which in turn dramatically increase resistance to these drugs.

To assess the clonal relationship between *aac(6')-Ib-cr*-positive isolates, the "enterobacterial repetitive intergenic consensus" (ERIC)-PCR genomic DNA profiles was analysed with specific primers [5]. Three different main subgroups were identified (Figure 1B). Most *aac(6')-Ib-cr*-positive samples were clonally related (82%), while 1 (6%) (#23) and 2 (12%) (#39 and #44) samples belonged to two different subgroups (Table 1).

We have shown for the first time the presence of the *aac(6')-Ib-cr *gene limited to *E. coli *species in North-East Italy. Like other plasmid-mediated resistance genes, i.e. *qnr*, the *aac(6')-Ib-cr *gene does not significantly increment MIC values, but it seriously increases the mutant prevention concentration (MPC) with final production of remarkably resistant strains upon treatment with standard FQ dosage [1]. Indeed, we found that all but one clinical isolate presented three mutations at the topoisomerase level (2 in GyrA and 1 in ParC) with consequent generation of very resistant strains (MIC of ciprofloxacin ≥ 32). These mainly derived by clonal expansion, as demonstrated by ERIC subgrouping. Interestingly, sample #44 did not show any mutation in the topoisomerase genes, and so retained full susceptibility to FQs. However, #44 resulted clonally related to sample #39 which instead was fully resistant, indicating that transition from susceptibily to resistance probably occurred in a very limited time interval.

Finally, *aac(6')-Ib-cr*-positive strains, which were strongly associated with ESBL, were collected mainly by inpatients and the proven plasmid localization and conjugation underline a very efficient mechanism of horizontal transferability of these multiresistant strains. Therefore, the presence of *aac(6')-Ib-cr*-positive strains must be promptly detected and referred to clinicians in order to avoid use of FQs which would augment drug resistance and impair therapy.

## Competing interests

The authors declare that they have no competing interests.

## Authors' contributions

IF carried out the molecular genetic studies and plasmid analysis. AC participated in the design of the study and in the selection of the clinical strains. CB performed the phenotypic analysis. SNR conceived of the study and participated in its design and coordination and drafted the manuscript. GP conceived of the study and participated in the coordination of the study. All authors read and approved the final manuscript.
